# Typology of adults diagnosed with mental disorders based on socio-demographics and clinical and service use characteristics

**DOI:** 10.1186/1471-244X-11-67

**Published:** 2011-04-20

**Authors:** Marie-Josée Fleury, Guy Grenier, Jean-Marie Bamvita, Michel Perreault, Jean-Caron

**Affiliations:** 1 Department of Psychiatry, McGill University, 845 Sherbrooke Street West, Montreal, Quebec, Canada, H3A 2T5; 2 Douglas Hospital Research Centre, 6875 LaSalle Boulevard Montreal, Quebec, H4H 1R3, Canada

## Abstract

**Background:**

Mental disorder is a leading cause of morbidity worldwide. Its cost and negative impact on productivity are substantial. Consequently, improving mental health-care system efficiency - especially service utilisation - is a priority. Few studies have explored the use of services by specific subgroups of persons with mental disorder; a better understanding of these individuals is key to improving service planning. This study develops a typology of individuals, diagnosed with mental disorder in a 12-month period, based on their individual characteristics and use of services within a Canadian urban catchment area of 258,000 persons served by a psychiatric hospital.

**Methods:**

From among the 2,443 people who took part in the survey, 406 (17%) experienced at least one episode of mental disorder (as per the Composite International Diagnostic Interview (CIDI)) in the 12 months pre-interview. These individuals were selected for cluster analysis.

**Results:**

Analysis yielded four user clusters: people who experienced mainly anxiety disorder; depressive disorder; alcohol and/or drug disorder; and multiple mental and dependence disorder. Two clusters were more closely associated with females and anxiety or depressive disorders. In the two other clusters, males were over-represented compared with the sample as a whole, namely, substance abuses with or without concomitant mental disorder. Clusters with the greatest number of mental disorders per subject used a greater number of mental health-care services. Conversely, clusters associated exclusively with dependence disorders used few services.

**Conclusion:**

The study found considerable heterogeneity among socio-demographic characteristics, number of disorders, and number of health-care services used by individuals with mental or dependence disorders. Cluster analysis revealed important differences in service use with regard to gender and age. It reinforces the relevance of developing targeted programs for subgroups of individuals with mental and/or dependence disorders. Strategies aimed at changing low service users' attitude (youths and males) or instituting specialised programs for that particular clientele should be promoted. Finally, as concomitant disorders are frequent among individuals with mental disorder, psychological services and/or addiction programs must be prioritised as components of integrated services when planning treatment.

## Background

Mental disorder is one of the leading causes of morbidity worldwide. Its cost and negative impact on productivity are substantial. Consequently, improving mental health-care system efficiency - especially service utilisation - is a priority. A systematic literature review reveals that prevalence rates at 12 months and lifetime are as follows: 10.6% and 16.6%, respectively, for anxiety disorders [1]; 4.1% and 6.7% for major depressive disorders [2]; 6.6% and 13.2% for alcohol use disorders; and 2.4% both in the case of drug use disorders [3]. Mental disorders are frequently associated with alcohol or drug use disorders. The U.S. National Comorbidity Surveys evaluated that 42.7% of respondents with alcohol or drug disorder also had a mental disorder in the 12 previous months, and 14.7% a mental disorder along with alcohol or drug disorder [4].

Risk factors and correlates to mental or substance use disorders have also been extensively investigated [5-8]. Age, gender, income, and marital and employment status are the principal socio-demographic factors associated with the presence of mental disorder. Being female, middle-aged, widowed, separated or divorced and a low-income earner increases the risk of major depressive disorder [6]. A systematic literature review showed that anxiety disorders were approximately twice as prevalent among females [1]. For substance use disorders, studies reveal a generally greater prevalence among males and youths [3].

Mental health-care service use has also been the subject of many epidemiological studies. The most frequently used model for identifying factors associated with service use is Andersen's behavioural model which classifies predictors of service use into three categories: predisposing, enabling, and needs-related factors [9]. Predisposing factors are individual characteristics that existed prior to the illness such as age, gender, language, marital status, race/ethnicity, and country of birth. Several studies have found that people aged 25 to 44 [10-12], females [10-17], previously married [12,15,16,18,19], highly educated [18,20], white [11,15,21], and native-born [22,23] are most likely to use health-care services. Enabling factors refer to features that influence care delivery and attitudes toward care; they encompass variables such as income, social support, and geographical location. The most important enabling factor is income. People with more elevated socio-economic status tend to use psychiatric and psychological care more assiduously, even among individuals with the same insurance coverage [20,24-26]. Finally, needs-related factors include assessments of physical and mental health by patients and professionals, including diagnosis, severity of the disorder, and perceived needs. Depressive disorders [20] and anxiety, particularly panic disorders [27,28] are strong predictors of health service use.

Utilisation of services has also been studied with regard to the use of primary mental health-care (e.g. general practitioners) or specialised mental health-care (e.g. psychiatrists). Individuals who use primary care are mainly female, older, more highly educated, live with a spouse or partner, and generally have anxiety or depressive disorders [29]. Conversely, frequent users of psychiatric services (second- or third-line services) are generally male, young or middle-aged, unemployed, live alone, have low social support and, often, a dual diagnosis of mental and substance use disorder [13,30,31]. Finally, youths and people with substance use disorders only use few health-care services [32,33].

DSM-IV, the most widely recognised mental health classification system, provides a detailed clinical profile of mental disorder; however, its use in forecasting needs or health-care service utilisation is limited [34]. An alternative classification suggests that mental health-care users can be described in terms of clusters based on several characteristics. With the use of clusters, persons may be considered in broad terms; in addition, subgroups may be correlated with clinical and socio-demographic variables and patterns of service use [34].

Cluster analysis has mainly been used to create typologies of patients with serious mental disorders [34]. Studies have identified frequent users of in-patient services [35,36], patients with schizophrenia treated in the community [37], patients with serious mental illness according to their level of functioning [38], patients with dual diagnoses of serious mental disorders and substance use [39] first-ever admitted psychiatric in-patients [40]. Very few studies have explored subgroups of individuals with common mental disorders. An exception is a study by Mitchell and colleagues [41] that used cluster analysis to classify adults with problematic online experiences and conventional problems (mental and physical health problems; family and/or other relationship problems; victimisation; aggressive behaviour) from a clinical perspective. Identifying individuals with common socio-demographic and clinical characteristics and patterns of service use, however, is essential to the efficacious planning of mental health-care delivery [38].

In an effort to enhance knowledge of service needs profiling, this Canadian urban catchment area study (258,000 persons served by a psychiatric hospital) includes a typology of individuals diagnosed with mental disorder in a 12-month period based on their individual characteristics and use of services. Variables used in the cluster analysis are based on Andersen's behavioural model [9], which considers that health-care service use is determined by predisposing, enabling, and needs-related factors.

## Methods

### Design and study population

The study focuses on an epidemiologic catchment area in the south-western section of Montreal, Canada. This area has a population of 258,000 and encompasses a broad range of social structures, socio-economic status, education, availability of health-care services, neighbourhood dynamics, and levels of security [42].

The catchment area includes six neighbourhoods, ranging in population from 23,205 to 90,640. Immigrants represent 25% of the population (versus 26% in Montreal). The proportion of low-income household represents 33% (versus 23% in the province of Quebec and 35% in Montreal). Low-income households are located mainly in two of the six neighbourhoods where close to half of the residents are low-income earners. Mental health-care services are chiefly delivered by three organisations: two health and social service centres (created through the merger of a general hospital, community local service centres, and nursing homes) that provide primary and specialised health-care services; and a psychiatric hospital that delivers specialised care (i.e. second- and third-line services). Sixteen community-based agencies (voluntary sector) offering primary mental health-care services are also present; they provide numerous services (e.g. crisis centre, day centres, self-help groups, back-to-work programs) to people with mental disorder or their relatives. General practitioners and psychologists practising in private clinics complete the primary mental health-care system in this area.

### Selection criteria and sample

To be included in the survey, participants had to be aged 15 to 65 and reside in the study area. The sampling was equally distributed in the study area among the various neighbourhoods [42]. The discrepancy between the study population and the sample has been readjusted as regards age and gender distribution by allocating a thoroughly calculated weight to each participant.

Interviews were conducted at home using portable computers. Only one person per target household was selected using procedures and criteria contained in the *National Population Health Survey *(NPHS, 2003-2005). Participants had the option to choose the language (English or French) of their interview. The research was approved by the Douglas Hospital Research Ethics Board Committee. Data were collected in a random sample of the catchment area from June to December 2009 by specially trained interviewers. Each participant was required to sign a consent form before answering the questionnaire. For those aged 15 to 17, parents had to give authorization before the interview.

A randomly selected sample of 2,433 individuals took part in the survey. The mean age of the sample was 42.4 (SD: 13.3). Sixty-three percent were female. After weighting for age and gender, the mean age of the sample was 40.7 (SD: 14.1) and the proportion of females was 52%. Forty-five percent were married or common law versus 17% divorced or separated and 37% single. Seventy-two percent completed post-secondary education and 77% held a job in the last 12 months. French was the first language for 54% of participants and English for 22%. Eighty-two percent were Caucasian; 24% were non-European immigrants. Average personal income was CA$28,688 (SD: 31,061) and average household income, CA$49,566 (SD: 51,057). A full description of the study has been published [42].

### Variables, measurement instruments and data collection

Variables assessed in the descriptive analysis (and, in part, in the cluster analysis) are displayed in Table 1. Variables are categorised according to Andersen's behavioural model for predisposing factors, enabling factors, and needs-related factors and health-service utilisation [9]. According to the literature, the most influential predisposing factors are age, gender, marital status, household size, and education. Also included among predisposing factors in this study were first language and country of birth, since linguistic or cultural differences can be barriers to health-service access. The most important enabling factor is income (household and main source). Needs-related factors include type and number of mental disorders and psychological distress. Finally, health-service utilisation includes services provided according to types of organisation (i.e. primary or specialized care) and professionals consulted (e.g. psychiatrists, psychologists, and general practitioners).

**Table 1:** Variables assessed in the study

Variables			Measuring Instruments
**Predisposing factors**	Socio-demographic variables^1^	Age	
		Gender	
		Marital status	
		Household size	CCHS 1.2 (Statistics Canada 2001)^a^
		Education	
		First language	
		Country of birth	

**Enabling factors**	Economic factors^1^	Income (household; main source)	CCHS 1.2 (Statistics Canada 2001)^a^

**Needs-related factors**	Mental disorders (type and number)		Composite International Diagnostic Interview (CIDI), (Statistics Canada 2000)^a^
			Drug Abuse Screening Test (DAST)^a^
			
	Psychological distress^5^		Alcohol Use Disorders Identification Test (AUDIT)^a^

**Health-service utilisation**	Services provided in hospitals (including hospitalisation), mental health centres, rehabilitation centres, private clinics, pharmacies, and in the voluntary sector (e.g. support groups, crisis-line services).		
	Professionals consulted: psychologists, general practitioners, psychiatrists, case managers, toxicologists, nurses, social workers, psychotherapists, pharmacists, other health professionals.		

^a ^Measurement instruments validated in the French-speaking population

Many instruments were used to measure specific health and psychosocial parameters. Mental health diagnostics are based on the Composite International Diagnostic Interview (CIDI), an instrument created by a WHO working group [43]. CIDI diagnoses, based on DSM-IV, include anxiety disorders (e.g. agoraphobia, social phobia, panic disorder), mood disorders (major depression, mania) and substance-use disorders (alcohol and drug abuse and dependence). Since its development in 1990, the CIDI has been used in several large-scale community epidemiological surveys throughout the world [43-46]. Substance abuse level was measured with the Drug Abuse Screening Test (DAST), a 20-item (yes/no) measure of past-year drug use [47]. Alcohol use level was assessed with the Alcohol Use Disorders Identification Test (AUDIT), a ten-item questionnaire (yes/no) measuring the degree of dependence and high-risk alcohol consumption [48]. Psychological distress was measured with the k-10 psychological Distress Scale [49], which contains 10 questions assessing the frequencies of previous recent psychological distress on 5-point Likert scale [50]. Socio-demographic and economic data were collected using the Canadian Community Health Survey questionnaire (CCHS 1.2). The questionnaire on mental health service use was adapted from CCHS 1.2. Participants who were identified with mental or emotional problems were invited to indicate the services they used, type and frequency of utilisation, and degree of appropriateness of the service they used. Services covered by this questionnaire were those offered in hospitals (including hospitalisation), mental health local community service centres, rehabilitation centres, private clinics, pharmacies and by the voluntary sector (e.g. support groups, crisis line services), including the following professionals: psychiatrists, psychologists, general practitioners, case managers, toxicologists, nurses, psychotherapists, pharmacists and other health professionals. All of these instruments were validated among the French-speaking population.

### Analyses

Frequency distributions were calculated for categorical variables. For continuous variables, mean values and their standard deviations were generated. Eleven variables were selected for cluster analysis, based on their impact on service use and the potential to characterise user profiles. The clustering of participants, based on individuals who were diagnosed with mental disorders, was computed using SPSS Statistics 17.0. The only multi-categorical qualitative variable was 'age'. Other categorical variables were: gender, alcohol dependence, drug dependence, anxiety (panic disorder, agoraphobia, social phobia), major depressive disorder, and mania. Continuous variables were: household income, psychological distress score, number of mental disorders per subject, and number of health services used. Categorical variables were entered first, followed by continuous variables. To determine inter-subject distance, the Log-likelihood method was employed. Participants' classification was made using the Schwarz-Bayesian criteria. The final number of clusters was automatically determined according to their overall contribution to inter-class homogeneity.

## Results

### Description of the sample: predisposing, enabling and needs factors

Among the 2,433 people who took part in the survey, 406 (17%) experienced at least one episode of mental disorder in the 12 months pre-interview according to the Composite International Diagnostic Interview (CIDI) and were selected for the analyses described below. The sample was representative of the source population. The distribution characteristics of participants who were diagnosed with mental disorder are displayed in Table 2. With respect to *predisposing factors*, the sample consisted of 56% females. The mean age was 39.4 years (SD: 13.1). Eighty-one percent reported Canada as their country of birth. Most participants (51%) were single or never legally married. Regarding *enabling factors*, a large minority of participants (45%) earned a salary as their main source of income. Thirteen percent reported receiving social welfare, and 3% unemployment insurance. The mean household income, as shown in Table 3, was CA$43,650 (SD: $38,179). Regarding *needs-related factors*, the three most reported mental disorders in the 12 months pre-interview were major depressive episodes (52%), alcohol dependence (24%) and social phobia (20%). The mean score for psychological distress was 15.7 (SD: 7.8), and the mean number of mental disorders per subject, 1.47 (SD: 0.83).

**Table 2:** Frequency distribution of participants with mental disorders in the 12 months pre-interview (N = 406; data weighted as to age and gender)

			n	%
**Predisposing factors**	Gender	Female	229	56.4
		Male	177	43.6
	
	Age categories [n (%)]	15-24 years	70	17.2
		25-34 years	91	22.3
		35-44 years	92	22.7
		45-54 years	91	22.3
		55-69 years	63	15.4
	
	Education	Less than secondary school graduation	78	19.1
		Secondary school graduation, no post-secondary education	60	14.8
		Some post-secondary education	43	10.7
		Post-secondary degree/diploma	225	55.4
	
	Marital status	Never legally married (single)	207	51.1
		Legally married (and not separated)	66	16.1
		Separated (but still married)	15	3.8
		Common-law	58	14.4
		Divorced	49	12.0
		Widowed	11	2.6
	
	Place of birth	Canada	329	81.0
		Other	68	15.1
	
	First language	French	263	64.8
		English	135	33.2
		Other	8	2.0

**Enabling factors**	Main source of income	Salary	183	45.1
		Social welfare	52	12.8
		Rent or retirement pension	19	4.7
		Unemployment insurance	11	2.7
		Other	17	4.1

**Needs-related factors**	Type of mental disorder in the 12 months pre-interview	Major depressive disorder	209	51.5
		
		Dependence		
		Alcohol dependence	97	23.9
		Drug dependence	77	19.0
		
		Anxiety disorders		
		Social phobia	80	19.7
		Panic disorder	44	10.8
		Agoraphobia	29	7.1
		
		Mania	45	11.1
		PTSD	18	4.4

**Health-service utilisation**	Participants who have used health-care services		212	52.2

**Table 3:** Descriptive statistics of participants with mental disorders in the 12 months pre-interview (N = 406; data weighted as to age and gender)

		Minimum	Maximum	Mean	SD
**Predisposing factors**	Age	16	69	39.40	13.11

	Household size	1	7	2.00	1,18

**Enabling factors**	Total household income	0	228,000	43,650.03	38,179.43

**Needs-related factors**	Psychological distress score	1.00	37.00	15.6755	7.75732
	Number of mental health disorders per subject	1	6	1.47	0.683

**Health-service utilisation**	Number of health-care services used in the 12 previous months	0.00	8.00	1.8715	1.38426

### Health-service utilisation

Among the 406 participants who experienced at least one episode of mental disorder in the 12 months prior to the interview, 212 (52%) reported using health-care services for mental health reasons at least once. These 212 participants were beset mainly by major depressive episodes (N = 129; 61%). The mean number of health-care services used per subject in the same period was 1.9 (SD: 1.4). A majority of the participants (N = 111; 52%) used both primary and specialised mental health-care, as against 27% (N = 57) who used only primary care and 21% (N = 44) only specialised care. The professionals most often consulted by the 212 participants for mental health reasons were general practitioners (N = 134; 63%), psychiatrists (N = 122; 58%) and psychologists (N = 68; 32%). The majority of participants who sought help from psychologists (N = 39/68; 63%) had private health insurance. Forty people (19%) consulted four different types of professionals or more.

### Mental health user profiles: cluster analysis

Among the 406 participants, 222 (53%) were automatically clustered in four subgroups (Table 4), as regards their socio-demographic characteristics, mental health status, and health-service utilisation.

**Table 4:** Cluster analysis of participants according to socio-demographic characteristics, mental health disorder, and health-care service utilisation (N = 222; data weighted as to age and gender)

	Variables	Clusters				
		**1 [N = 57 (25.7%)]**	**2 [N = 45 (20.3%)]**	**3 [N = 73 (32.9%)]**	**4 [N = 47 (21.2%)]**	**Combined [N = 222 (100%)]**

Socio-demographic characteristics	Male [n (%)]	6 (9.1)	17 (25.8)	20 (30.3)	23 (34.8)	66 (100)
	Female [n (%)]	51 (32.7)	28 (17.9)	53 (34.0)	24 (15.4)	156 (100)
	Age categories [n (%)]					
	15-24 years	0 (0)	13 (59.1)	0 (0)	9 (40.9)	22 (100)
	25-34 years	18 (40.0)	7 (15.6)	10 (22.2)	10 (22.2)	45 (100)
	35-44 years	20 (29.9)	17 (25.4)	18 (26.9)	12 (17.9)	67 (100)
	45-54 years	11 (19.6)	6 (10.7)	29 (51.8)	10 (17.9)	56 (100)
	55-69 years	8 (25.0)	2 (6.3)	16 (50.0)	6 (18.8)	32 (100)
	Household income [mean (SD)]	37,408.50 (37,070.10)	27,486.10 (23,895.20)	47,359.00 (42,396.70)	30,869.70 (34,131.60)	37,284.90 (36,766.90)

Mental health disorders in the 12 previous months	Psychological distress score [mean (SD)]	16.8 (7.9)	19.1 (7.3)	15.3 (7.9)	13.9 (7.3)	16.2 (7.8)
	Alcohol dependence [n (%)]	1 (2.3)	15 (34.9)	0 (0)	27 (62.8)	43 (100)
	Drug dependences [n (%)]	0 (0)	19 (52.8)	0 (0)	17 (47.2)	36 (100)
	Anxiety (panic disorder, agoraphobia, social phobia) [n (%)]	57 (77.0)	17 (23.0)	0 (0)	0 (0)	74 (100)
	Major depressive disorder [n (%)]	28 (20.9)	33 (24.6)	73 (54.5)	0 (0)	134 (100)
	Mania [n (%)]	0 (0)	24 (100)	0 (0)	0 (0)	24 (100)
						
	Number of mental disorders per subject [mean (SD)]	1.7 (0.7)	2.6 (1.1)	1.0 (0.1)	1.1 (0.2)	1.5 (0.8)

Health-care service utilisation in the 12 previous months	Number of health services used [mean (SD)]	2.6 (2.1)	3.0 (2.2)	2.4 (1.6)	2.1 (1.9)	2.5 (1.9)

Label		Young females with anxiety disorders	Young low-income earners with multiple mental and dependence disorders	Middle-aged, high-income females with depressive disorders	Young low-income earners with dependence disorders	

Cluster 1 comprised 57 users out of 222 (26%), predominantly persons between 25 and 44 years of age (38/57 or 67%). The prototypical member (51/57 or 89%) was a female affected by anxiety disorders (panic disorder, agoraphobia, social phobia). Half also experienced a major depressive episode in the previous 12-month period. Only one member of this cluster was affected by alcohol dependence and none by drug dependence or mania. This cluster ranked second with respect to household income, psychological distress score, number of mental disorders per subject, and number of health-care services used. It ranked third as to proportion of people with major depressive episodes. Participants in this cluster may be characterised as 'Young females with anxiety disorders'.

Cluster 2 comprised 45 users (20% of the sample) and included a majority of younger participants (15-24 years) (13/22 or 59%). Males were over-represented in this cluster (17/45 or 38% vs. 66/222 or 30% for the sample as a whole). The prototypical member of Cluster 2 was more frequently beset with mental disorder than all other users, with a mean of 2.6 per subject. This cluster encompassed all cases of mania, the greatest proportion of drug dependence, and the highest mean psychological distress score. It ranked second with regard to the proportion of alcohol dependence and anxiety disorders. It ranked first for the mean number of health-care services used. Finally, participants in this cluster reported the lowest household income. They may be referred to as 'Young low-income earners with multiple mental and dependence disorders'.

Cluster 3 comprised 73 users (33% of the sample) who were predominantly older (45/73 or 62%) (45-69 years). The prototypical member of this cluster was a female (53/73 or 73%) with elevated household income and affected exclusively by major depressive disorder. None was diagnosed in the last 12 months with mania, anxiety, drug or alcohol dependence. This cluster ranked third as to psychological distress and number of health-care services used, and fourth with regard to the number of mental disorders per subject. Participants in this cluster may be characterised as 'Middle-aged high-earning females with depressive disorders'.

Finally, Cluster 4 comprised 47 users (21% of the sample). This cluster featured the most evenly distributed age categories. It was also the only one where males (49%) and females (51%) were almost equally represented. The prototypical member of this cluster was a person affected predominantly by alcohol and/or drug dependence but not by anxiety or mood disorders. Cluster 4 ranked third as to household income and number of mental disorders per subject. It ranked fourth with respect to the number of health-care services used and psychological distress. Participants in this cluster may be called 'Young low-income earners with dependence disorders'.

## Discussion

The study was designed to develop a typology of individuals, diagnosed with mental disorder during a 12-month period, based on individual characteristics and use of services. Its purpose is to generate knowledge on service needs profiling in support of efforts to facilitate mental health-care service planning. Mean prevalence of mental disorder in the last 12 months was 17%. According to epidemiological studies, the prevalence of mental disorder varies widely from country to country. In the International Consortium in Psychiatric Survey (ICPE), which focused on seven countries, the overall prevalence at 12 months was 29% in USA and 20% in Canada, as against 8% for Turkey [43]. In a recent study based of a sample of more than 21,000 adults representative of the overall population in six European countries, Alonso and Lépine [51] estimated the proportion of people affected by a mental disorder in the 12 previous months to be 11.5%. More recently, a meta-analysis of 27 studies estimated at 27% the proportion of European adults with at least one mental disorder in the 12 previous months [52]. Differences in survey methods or instruments may account in part for these considerable variations [5,51,53]. Some disorders (e.g. bipolar disorders and drug dependence in Western European countries) were not assessed in all the surveys [53]. A greater reluctance toward participation or admission of mental illness in some countries and/or interviewer error are other possible biases that can explain the under- or over-estimation of the prevalence of mental disorders [53,54].

In our study, almost 50% of persons affected in the last 12 months by a mental disorder used health-care services for mental health reasons. In comparison to other studies, this number is high. According to the 2002 Canadian Community Health Survey Mental Health and Well-Being (CCHS 1.2), only 38.5% of Canadians used services for mental health reasons, when at least one mental disorder was present [55]. In the 1997 Australian National Survey of Mental Health and Well-Being, only 35% of people with at least one mental disorder sought professional help [56]. In our study, the proximity of a psychiatric hospital may account for the more assiduous use of mental health-care services. Globally, studies demonstrate that health-care services are underused by individuals with mental disorder. In Quebec and the rest of Canada, where public services are focused on the treatment of serious mental disorders and since psychological services are only partially available in the public health-care system, access to treatment for people with less severe disorders and without private insurance and/or with low income is limited. These situations explain in part the underutilisation of services for mental health reasons.

In our study, individuals who used services for mental health reasons received two services, on average, mainly from primary and specialised care providers, and close to 20% consulted at least four mental health-care professionals. The use of diverse and increasingly community-based professionals is perceived as a positive development by various authors [57,58]. Individuals who receive dual-modality treatment (e.g. psychopharmacology and psychotherapy) are less likely to abandon treatment than people who consult psychiatrists only [58]. However, individuals with low income do not have easy access to psychologists (a service that often requires private insurance). A recent study has shown that cost is the main obstacle to psychotherapy access, especially for people with anxiety disorders [59].

Cluster analysis yielded four user profiles including people with mainly anxiety disorders (Cluster 1), depressive disorders (Cluster 3), alcohol and/or drug disorders (Cluster 4), and multiple mental and dependence disorders (Cluster 2).

Two clusters (1 and 3) were more closely associated with females. In the other clusters (2 and 4), males were over-represented in comparison with the sample as a whole. It is interesting to note that these two clusters (where males are a majority) are linked to dependence disorders, regardless of association with mental disorders. The socio-demographic variables associated with the various clusters were consistent with previous studies on the prevalence and correlates of anxiety, mood, and dependence disorders in the general population. Our results show that anxiety [1] and depressive disorders [6] are more prevalent among females, and dependence disorders, principally alcohol dependence, and concurrent disorders more common among males [3].

In three clusters, users aged 34 years or less were over-represented. Young adulthood is a critical life stage at which people leave the family home and may make far-reaching decisions with respect to education, career or parenthood [60]. Generally, mental disorders begin during this period [7]. Most general population surveys have found a marked prevalence of mental disorder in young adulthood. For example, in the National Comorbidity Survey Replication study (NCS-R), three-quarters of lifetime mental disorders emerged by age 24 [60]. Youth is also associated with a greater risk of hospital readmission [13,61]. Conversely, age is a protective factor, with the risk of mental disorder decreasing as one gets older [62].

Age of onset may account for the differences between Clusters 1 and 3. Cluster 1 included young women with anxiety disorders for the most part, though one half did experience a major depressive disorder. Conversely, Cluster 3 includes mainly middle-aged women who had only one major depressive episode without anxiety disorder in the last 12 months. It is possible that users in Cluster 3 successfully negotiated young adulthood and entered the job market or started a family without experiencing anxiety disorder or were successfully treated for it. Another explanation is that the major depressive episode occurred recently. If anxiety disorder tends to occur among younger individuals, mood disorder (including major depressive episodes) tends to occur among older individuals [63]. According to one study [6], lifetime rates and the probability of major depressive disorder are higher among baby-boomers than younger adults.

Co-morbidity with mental disorder appears to be the norm [64,65]. In three of four clusters, users were affected by more than one mental or dependence disorder. In Cluster 2, co-morbidity with mood disorder, anxiety, and dependence disorder was very frequent. Several studies indicate a significant correlation between alcohol dependence and depression [66-68]. Intoxication by alcohol or drugs can induce symptoms similar to those of depressive disorder [66]. Some drugs can increase stress and provoke panic attacks or other anxiety disorders [4]. It is also known than several people with anxiety or mood disorder use alcohol or drugs as self-medication [69]. Users with dual diagnoses present a challenge for mental health and addiction services and generally have worse treatment outcomes [64,67]. Co-morbid disorders are generally more chronic than pure mental disorders, and treatment is less effective [4]. Cluster 4 was distinguished from Cluster 2 by the absence of mania, major depressive episodes, and anxiety. Cluster 4 was characterised by dependence disorder, combined with more marginal mental disorder, and exhibited a propensity for alcohol, rather than drug, use. Several studies have revealed that alcohol dependence is generally not as strongly associated with mental disorder as is drug dependence [64,67,70,71].

Clusters 1 and 2 were the most keenly affected by psychological distress. Conversely, Cluster 4, which encompassed only 1.1 mental or dependence disorders per subject, was not as greatly impacted by psychological distress as other clusters. These results seem to confirm that the number of mental disorders is associated with greater psychological distress [64]. We may assume that multiple mental or dependence disorders can affect several domains essential to quality of life (work, daytime activities, social and intimate relationships, physical health, etc.).

Finally, Clusters 1 and 2 featured the greatest number of mental disorders per subject and the most frequent use of mental health-care services. According to several studies, needs factors are the prime predictors of service use [9,72,73], and greater numbers of mental disorders result in more frequent use of health-care services [64]. In addition, some authors have suggested that users with multiple mental and dependence disorders feel a greater impetus to seek treatment [64,74]. However, it is interesting to note that the mean number of health-care services used by Cluster 3, with only one mental disorder, is quite similar to that of Cluster 1. One possible explanation is that participants in Cluster 3, with the highest household income, make more frequent use of private psychologists to treat major depressive disorders. For Vasiliadis and colleagues, depression is the most significant predictor of service use [20]. Gender may also account for similarities between Clusters 1 and 3 regarding service utilisation. It is known that females use more health-care services in general, mainly general practitioners and other primary care services. Age may also explain the differences: middle-aged persons are the peak users of mental health-care services [75]. Conversely, younger people are less likely to perceive their need for treatment and often wish to solve problems on their own [32]. Young adults are also more likely to drop out of treatment [58]. Finally, participants in Cluster 4 use relatively few health-care services. This is the case for people affected only by substance disorders [33]. They are usually less likely to think they need help than participants with co-morbid mental disorders [74].

This study has some limitations. First, information was not available on participants' physical condition. Several epidemiological studies have reported that people with mental health disorders or dependence disorders often also have significant physical disorders, such as hypertension, diabetes or epilepsy [76,77]. The presence of a physical disease may account for more frequent use of health-care services. Second, our study did not include the full spectrum of psychiatric disorders, e.g. schizophrenia and other serious mental disorders, organic mental disorders, sexual disorders, eating disorders, personality disorders, and intellectual deficiencies. Several studies have reported a prevalence of personality disorders with anxiety, depression or substance-use disorders. Near half the people with a current mood or anxiety disorder have at least one personality disorder [78]; identifying these disorders would have allowed us to refine our cluster analysis. Finally, the severity of mental disorder was not considered. Previous studies have reported that severe cases use more services than mildly severe or moderate ones [33].

## Conclusion

The study found considerable heterogeneity among socio-demographic characteristics, number of disorders, and number of health-care services used by individuals with mental or dependence disorders. Overall, there is significant underutilisation of mental health-care services with female consuming more services than men. When individuals sought services for mental health reasons, they generally saw more than one provider and used both primary and specialised mental health-care. As services are underutilised and mental disorders vary with regard to gender, age, and other characteristics, it is crucial to develop treatment modes or service programs that target specific mental disorder profiles. Our study reveals the existence of four subgroups of users with mental disorders: 'young females with anxiety disorders'; 'young low-income earners with multiple mental and dependence disorders'; 'middle-aged, high-income females with depressive disorders'; and 'young low-income earners with dependence disorders'. The second group exhibited the most socio-economic vulnerability and most frequent service utilisation.

Along with the need to target the four subgroups above with specific programs, our study highlights the relevance of focusing on younger individuals affected by multiple mental disorders or anxiety disorders concurrent with or without major depressive disorders. As concomitant problems are frequent among people with mental disorders, psychological services and/or addiction programs must also be taken into consideration as components of integrated programs or shared-care initiatives when planning treatment. In addition, as males seem to consult only when they suffer multiple mental and substance disorders, more outreach and promotion programs are needed to detect and facilitate mental health-care service access for them. Globally, public education on drug use and mental disorders and programs specially designed for youths and/or males may reduce this clientele's reticence to use health-care services. Integrating motivational and cognitive aspects of behavioural change in professional training may also lead to greater mental health-care utilisation. At last, enabling greater collaboration within the health-care system between professionals (including general practitioners) and programs, especially with regard to mental disorders and substance abuse, should lead to more timely and appropriate care.

## Competing interests

The authors declare that they have no competing interests.

## Authors' contributions

MJF, GG and MP designed the study. JMB carried out the statistic analyses with assistance from JC. MJF and GG wrote the article. All authors have read and approved the final manuscript.

## Pre-publication history

The pre-publication history for this paper can be accessed here:

http://www.biomedcentral.com/1471-244X/11/67/prepub
